# Introduction

**DOI:** 10.1111/1475-6773.13219

**Published:** 2019-10-24

**Authors:** Robert J. Blendon

**Affiliations:** ^1^ Department of Health Policy and Management Harvard T.H. Chan School of Public Health Boston Massachusetts

This Special Issue of *Health Services Research* on discrimination in America was conducted in the midst of a national debate on the extent of discrimination in the lives of Americans,^1^, ^2^, ^3^, ^4^, ^5^, ^6^, ^7^ from the perspectives of six under‐represented groups in public opinion research on discrimination: nationally representative samples of blacks, Latinos, Native Americans, Asian Americans, women, and lesbian, gay, bisexual, transgender, or queer (LGBTQ) adults. Despite strong evidence outlined in Williams et al in this issue and prior literature widely showing that discrimination has significant, harmful effects on health and well‐being,^8^, ^9^, ^10^, ^11^ public opinion is currently divided over whether discrimination facing minority and marginalized groups in America today exists or is a serious problem.^2^, ^3^, ^4^, ^5^, ^6^, ^7^ These questions, and the divide over the answers, are critical to both policy action to end discrimination in the United States and the health outcomes of marginalized populations. Are the numerous examples of real discriminatory behavior against members of minority and marginalized communities that appear in the media just tragic, isolated acts, or are they part of a much larger pattern of institutional discrimination and culturally biased behavior? If there are broad patterns of discrimination, are they focused just on one minority or marginalized community, or do they impact a broad range of Americans today?

The answers to these questions, as portrayed in each of the articles in this Special Issue, are fundamental to the experiences of millions of Americans and American residents, as well as to the US's future as an increasingly multiracial, multiethnic, and gender‐diverse nation. The results shown in this Special Issue also involve questions of fairness, equity, and safety. This study was conducted in the midst of growing racial divides in civil and political discourse in the United States,^2^, ^4^, ^5^, ^7^ alongside documented rises in reported hate crimes in America primarily motivated by race, ethnicity, religion, and sexual orientation.^12^ While civil and human rights groups have recently shown some federal policy progress on eliminating discrimination against minorities in key areas that affect their lives and health, they have also documented major gaps in policies and opportunities for the future across the sectors of health care, criminal justice, education, employment, housing, and voting.^1^ In addition, as shown by Williams et al in this issue,^8^ major patterns of discrimination can significantly harm the health and broader well‐being of minority and marginalized populations, even adding more importance to these questions.

The Robert Wood Johnson Foundation funded this project in partnership with NPR and Harvard TH Chan School of Public Health to bring a public health perspective to the complexity and pervasiveness of discrimination in the United States today. This polling effort extends the Foundation's work to build a culture of health in America by examining adults' experiences of discrimination, given the large body of research showing it is an important determinant of health.^8^, ^9^, ^10^, ^11^ In particular, this polling effort extends prior work in this area by (a) focusing on people's reports of their own direct life experiences with discrimination, rather than general perceptions of discrimination in the country broadly; (b) examining experiences of discrimination across a wide range of areas of life, including both institutional discrimination and more interpersonal or individual‐level discriminatory behaviors; and (c) simultaneously capturing the reported life experiences across six groups whose experiences are often overlooked because they are difficult to sample in telephone polling.

The health services research field is now part of a growing movement to broaden the vision of what constitutes “health services” beyond health care, to include the impact of social, physical, and economic forces on population health.^13^, ^14^, ^15^, ^16^, ^17^, ^18^ We believe new research on discrimination is essential for health services researchers' body of knowledge, as social determinants of health both within and outside of the health care system can have important long‐term impacts on patients' overall health and well‐being.^8^, ^9^, ^10^, ^11^ We wanted to alert health professionals and health services researchers to these problems among their patients, which extend beyond health care. We chose to examine discrimination in both health and nonhealth arenas, to highlight the extent of discrimination for health services researchers in a variety of areas that affect not only Americans' health care, but also their well‐being, safety, housing, and opportunities in education and the workplace. Future research should recognize that although there is a strong body of evidence establishing the need to narrow racial and ethnic disparities in health care,^19^ addressing how to narrow these disparities and end discrimination are extremely complex. New solutions need to go beyond frequently mentioned options, such as diversity training or quality improvement, which have limited evidence supporting their impact on discrimination reduction.

The issues covered by this polling effort involve over a dozen aspects of American life. Each of these issues carries its own ongoing debate about policies and programs that might improve life for minorities and marginalized adults by narrowing disparities and ending discrimination. However, achieving equity in areas as diverse as the workforce, policing, and voting is extremely complex, and there is no national consensus on how these problems should be solved. However, experts have recommended a myriad of different solutions worthy of serious consideration and study, including policies to improve socioeconomic opportunities for minority and marginalized populations, civil and human rights protections that do not exist or are not enforced in current US law, and the inclusion of ending discrimination as an explicit goal in research, policies, and programs.^1^, ^19^, ^20^ Our data suggest that more action is needed to reduce and eventually end discrimination in America, as most interventions aimed to discrimination reduction have not been rigorously evaluated for their effects on improving health outcomes or reducing health disparities. While it is beyond the scope of these results to make specific recommendations for how to end discrimination in each area of American life studied in this polling effort, this Special Issue provides important evidence that more research and practice on discrimination are sorely needed in health services research.

In presenting the results in each paper, we deliberately chose not to compare the experiences of various minority or marginalized groups to each other, nor did we compare the experiences of LGBTQ adults to non‐LGBTQ adults or women to men. We included whites as a comparison group to racial/ethnic minorities to ground self‐reported experiences in today's context and for consistency with prior research, but importantly papers focus primarily on each group of inquiry rather than on disparities against each other. We did this because the overall purpose of this polling effort was not to construct a hierarchy or rank ordering of discriminatory experiences, but rather to report directly from members of many minority or marginalized groups about their personal experiences with discrimination in the United States today. Wherever sample sizes allowed, we examined subgroups of some minority and marginalized populations, including Asian adults by East, Southeast, and South‐Asian heritage, racial/ethnic minority and white women, racial/ethnic minority and white LGBTQ adults, and transgender adults. By examining racial/ethnic minority groups, women, and LGBTQ adults separately in different papers, we focus on the unique experiences of each group and the ways in which discrimination, racism, and sexism have shaped their experiences in our contemporary moment. Taken together, this series of papers provides a powerful picture of discrimination in America today and raises deep concerns about these issues in the future if they cannot be addressed.

This polling effort extends prior research in building a base of recent evidence on reported experiences of discrimination, using national telephone polling data on groups who are often overlooked, to inform future policy debates over this issue. We believe the value of these polls is to provide reports of life experiences on hard‐to‐reach populations to complement ongoing national work on these issues.^1^, ^12^ It is often not realized that sampling minorities and other marginalized populations in polling and other probability‐based research can be extremely difficult, due to financial resources, technical, and cultural expertise needed. For example, even in sampling the Latino and Asian populations, we recognized from prior work these populations are quite heterogeneous, representing different geographic, ethnic, and cultural backgrounds.^21^, ^22^ LGBTQ adults are another important example of heterogeneity, as they have diverse racial/ethnic identities, sexual orientations, and gender identities that uniquely impact their lived experiences. It remains a major research challenge to examine adults' unique experiences within the larger LGBTQ population, particularly in probability samples.^23^ While we were able to make some distinctions in these papers, we were limited in some cases by sample sizes. In the future, researchers should recognize the difficulty in both sampling and generalizing findings within smaller, hard‐to‐reach, or diverse populations.

Within health care and beyond it, systemic change is needed to reduce and eliminate discrimination, because it carries such severe economic, social, and health consequences. The findings in these articles highlight that greater policy action is needed to create, implement, and evaluate major interventions and policies that address institutional patterns of discrimination in the United States today. Further change and research are needed to protect the civil and human rights of minority and marginalized populations, as well as to reduce health disparities and improve their health.
